# Factors Predicting Older Patients′ Family Involvement by Nursing Staff in Hospitals: The View of Hospital Nurses in Israel

**DOI:** 10.3390/healthcare10101921

**Published:** 2022-09-30

**Authors:** Dafna Halperin, Michal Mashiach-Eizenberg, Hedva Vinarski-Peretz, Nasra Idilbi

**Affiliations:** 1 Department of Community Gerontology, Yezreel Valley Academic College, Yezreel Valley 1930600, Israel; 2 Department of Health Systems Management, Yezreel Valley Academic College, Yezreel Valley 1930600, Israel; 3 M.A. Program in Public Administration and Public Policy, Department of Political Science, Yezreel Valley Academic College, Yezreel Valley 1930600, Israel; 4 Department of Nursing, Yezreel Valley Academic College, Yezreel Valley 1930600, Israel; 5 Nursing Research Unit, Galilee Medical Center, Nahariya 22100, Israel

**Keywords:** family-centered approach, family, nurses’ attitudes, involvement, nursing care, interactions, hospital, older people

## Abstract

According to the family-centered approach, the involvement of family in the care of hospitalized older patients is a crucial element of quality care. Active involvement of family in care by the nursing staff depends on different factors, including attitudes towards the importance of family in the care and perception of the interactions with the family. This study aims to identify the factors predicting staff behavior of involving the family in the care process. A cross-sectional study was conducted among 179 nursing staff at a hospital, using a self-report questionnaire examining staff attitudes towards the importance of family in care, the perception of the interactions with the family (family behavior, communication and conflicts), and staff behavior toward family involvement. The findings point out the importance that staff attitudes have on their behavior in the active involvement of family in the care of older patients. Staff behavior of family involvement was predicted by their perceptions of the family (as conversational partners and having their own resources), less conflicts with the family, and staff academic education. Staff behavior toward family is influenced by their attitude and staff–family relationships. Educational programs should emphasize the importance of family, as well as dealing with conflicts.

## 1. Introduction

Older people (65+) are a major section of the health system’s clients. They accounted for 35.7% of hospitalizations in the general hospital wards in Israel in 2019, and as much as 60% in Internal Medicine and Intensive Care wards [1]. During hospitalization, the nursing staff is officially responsible for caring for older patients, even if most of them are accompanied by family members who feel responsible for their relatives and provide at least some of the necessary support and care [2].

Much has been written about the importance of family in the decision-making and care process regarding older hospitalized patients, as part of holistic, health-promoting care. This necessitates cooperation between the medical and nursing staff and the patient’s family, but an up-to-date literature review shows that family and nursing staff have different positive and negative experiences related to families’ presence and ongoing involvement.

Ref. [3], which may impact nursing staff behavior. The present study examines the relation between nursing staff’s perception of the importance of family members caring for older patients and their interactions with the family, and the staff’s behavior regarding involvement of family in the care process, based on the family-centered approach.

### 1.1. Family Members during Hospitalization

Family members accompany older patients’ hospitalization in different hospital wards, regardless of the length of hospitalization [4,5,6,7]. The family is an extremely valuable resource in patient care, playing an important role in providing emotional support and patient recovery during hospitalization [8]. Families caring for their older relatives in the community pass the responsibility for their ongoing care to the hospital nursing staff during the relatives’ hospitalization [6], but at the same time continue to support and care for them [5,9]. The purpose of family involvement is for older patients to continue their lifestyle, preserve their dignity and promote their comfort level, allowing them to recuperate [9]. In addition, to preserve their well-being and quality of care, particularly when they are suffering from dementia [10]. Nursing staff perceive the emotional support of the patient as a joint responsibility with the family, while they see themselves as being more responsible for the daily physical care, including ADLs (Activities of Daily Living) [2].

### 1.2. Family Members’ Involvement: Factors and Characteristics

Family involvement in caring for hospitalized older people is comprised of different dimensions, such as regular visits, social-emotional support, advocacy, provision of personal care such as feeding, and participation in meetings planning continued care [6,11]. There may be different reasons for the family’s wish to be involved in this way, among them the perception that quality of care will be better, assuaging their guilt by visiting and connecting with the staff [12]; feeling committed to the patient and providing him/her with emotional and physical support [4]; mediation of information from the staff [11]; and providing a sense of security to the patient in an unfamiliar environment [13].

The family’s involvement also includes their own expectations regarding their place in the care process, receiving information, participating in decision-making, and communication with and receiving attention and support from the staff. They expect also for social and emotional involvement in the care process, as well as personal attention to both themselves and the patient by the staff, demonstration of warmth and respect, a polite demeanor, and sharing of information and decisions [14,15].

### 1.3. Staff–Family Members Interactions

The literature points at the importance of communication and a good relationship between the staff and the families in order to improve the quality of care [5,16,17]. Family members are a source of information regarding the older patient, their habits and needs, and can thus contribute to planning, decision-making and provision of better-quality appropriate care, as well as preserving the patient’s self-worth as much as possible [16,18]. Reliable working communication between the staff and the family has great significance in ensuring that the family is involved in care-related decision-making, as well as the planning of discharge from the hospital, as these predict a successful discharge for the patient [6]. However, the literature points to a wide range of staff–family relationships, from close proximity, openness and cooperation as colleagues in the care process, all the way to low-level relationships [14,17].

Different factors influence the creation of successful relationships, among them effective communication [12,14,17], joint understanding of both parties regarding the division of responsibility for different tasks [6,12], acceptance, respect and knowing that the others are there to help [19]. Moreover, the family’s perception that the staff accepts and respects their feelings, such as mourning or guilt, was found to contribute to this relationship [12,17]. Thus, the relationship and the family’s involvement depend, among other things, on the staff’s perception of the family, their involvement, their role and the communication with them.

### 1.4. Staff Members’ Perceptions Regarding Older Patients’ Family Members

The topic of staff–family relationships and their cooperation within different care frameworks has been amply researched in different countries in the last 20 years [16,18,19,20,21,22,23,24,25,26,27,28]. The research has dealt with staff and family perceptions and attitudes towards each other, their relationships and the factors which promote or hinder them.

The reports regarding staff perceptions and attitudes towards family members are not unequivocal and include positive, ambivalent, and negative attitudes [4,22,25,29]. On the one hand, families are perceived as an important and valuable resource, whose involvement and assistance are extremely appreciated, allowing the staff to lean on them [4,16,18,19,22,27]. This encourages their invitation to participate in caring for the family member and creates a good staff–family relationship [16,22,24]. A good relationship is characterized by the perception of families as partners in care, effective, open communication, participation in decision-making and provision of support [30,31]. Staff members who believed that family presence is important tended to involve the family more in the caring process [32].

On the other hand, it is possible to see more negative attitudes expressed by staff members towards families. Some of the families were perceived by staff members as being difficult to work with [12,30], while others were termed “problematic families” or “demanding families” [33,34] burdening the staff [4,22]. Other staff members perceived the families as too needy, disruptive, or inappropriately involved in the lives of older people living in an institution [31]; or conversely, they thought that the families do not do enough for the hospitalized person and are too self-involved [4,29]. Staff–family interactions were described as difficult, problematic, time-consuming, conflictual or challenging [25,33,34].

Thus, despite the common goal of providing the older patient with quality care, staff–family relationships can be difficult, with the interactions and communication being a source for tension and conflicts between them [31,35,36], leading to lack of cooperation, burnout among the nursing staff and dissatisfaction among family members.

### 1.5. Factors Related to Staff Perception of Family Members

Different factors, personal or connected to family members, may be related to staff perceptions of family members, the importance of their involvement in the care process and staff behavior towards them. All these may promote or hinder the creation of a good relationship between the parties. Basic staff perception of family members as guests, visitors or partners in the care process affects their perception as a source of support or hindrance [37]. Perceptions of family members as useless as care partners [38], or frail and vulnerable [7], as well as feelings of an inability to provide a response to the family’s demands [38], may act as barriers for involving the family in the care process.

In addition, disagreement between staff and family regarding the older person’s care needs [35] contributes to the conflicts. Staff suspicion that the family’s deep involvement is based on a lack of trust in their capabilities also contributes to the conflicts and lack of wish to cooperate [39].

Conflicts between nursing staff and family members may adversely affect the work performance of the staff, their well-being and sense of security in keeping the patient safe [20]. As conflicts with family members routinely occur in the health system [34,35,39], they may promote burnout and dissatisfaction among the staff, and influence their motivation to encourage family involvement.

Nurses’ personal characteristics, such as age, education, professional seniority and organizational seniority, were found to be connected to their perception of family involvement, or conversely, to discomfort regarding including family members in the care process [7,21,26,28]. Perception of family behavior is also connected to staff perceptions and motivation to create this connection. For example, assisting the staff, feelings of trust and providing information regarding the patient, his/her medical history and the care s/he had received were perceived as positive, as they save the staff time, allowing them to provide the patient with swift, appropriate care [16,25]. However, there are different reasons for a negative perception of the family, such as lack of cooperation [38], family members’ high, sometimes unrealistic, inconsistent and ambiguous expectations, or a feeling that they had not received an appropriate response [4,36], complaints or numerous demands made by family members towards the staff, and expressions of criticism, anger and hostility [11,33,34]. This may cause a barrier or hinder cooperation and the forming of a connection, as well as becoming a source of conflict [39]. Ethnic and cultural aspects may also have an effect. Family members with cultural beliefs and preferences different from those of the staff make things harder for the latter. Communication is more complex, due to the differences in beliefs, traditions and language, so a lack of understanding and different interpretations constantly take place, making it difficult to provide the family with information [4].

### 1.6. The Family-Centered Approach

A theoretical and practical framework of the family-centered approach has developed providing discourse regarding caring for older hospitalized patients [40,41,42]. The approach is based on the belief that the family has a significant effect on the patient’s health and mental welfare, and may contribute to and participate in planning and care with professional caregivers [43]. Thus, the patient and his/her family are treated as one unit [44,45]. The approach includes three basic concepts: respect, cooperation and support [45,46], as well as central characteristics, such as the importance of communication, patient and family involvement, support for the family and the importance of the nursing staff attitudes [47]. Care based on this approach includes, above and beyond treatment with respect, also taking into account the patient’s and family’s experiences, preferences and culture, with these forming the communication and the relationships between them and the staff [42].

The family-centered approach emphasizes the family’s active cooperation regarding patient care, decision-making and discussions of care preferences [48], and following this the importance of the relationship between the professionals and the family, taking into account their choices [44]. This requires commitment by the professionals, effective communication with the patient’s family, creating a good relationship with them, encouraging them to be actively involved in patient care, and sharing information and the decision-making process with them [42,43,48].

This literature review emphasizes the importance of including the patient’s family in his/her care during the hospitalization period. To the best of our knowledge, there is very little research examining actual nursing staff behavior in relation to their perception of the importance of family involvement as a meaningful factor in the family-centered approach. The research question of the present study is–What is the effect of the nursing staff perception of the importance of family in the care process and the interactions with them (family behavior, communication and conflicts) on the staff behavior regarding their involvement of family members in care?

### 1.7. Research Aims

Examination of the correlations between perceptions and individual factors, and staff behavior regarding involvement of family members in patient care.

Identification of the factors predicting staff behavior regarding involvement of family members in patient care.

**Figure 1:**
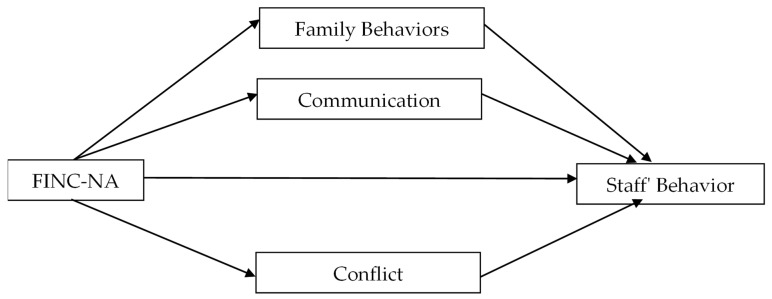
The research model. Abbreviation: FINC-NA, Families’ Importance in Nursing Care–Nurses’ Attitudes.

This article presents one of several models tested in a wider study. The model used in the current study is presented in Figure 1.

## 2. Materials and Methods

### 2.1. Study Design

This study used a cross-sectional correlational design, specifically a closed questionnaire administered to a convenience sample of 193 nurses in a hospital. Ethical approval was obtained prior to data collection from the Yezreel Valley College Ethics Committee.

### 2.2. Participants and Data Collection

A convenience sample of 193 nurses from 13 Internal Medicine, Surgical and Geriatric wards at a large hospital in the north of Israel.

Data collection was conducted from the beginning of March to the end of May 2022. One of the researchers, working at a large hospital in northern Israel, personally approached the nursing staff in the Internal Medicine, Surgical and Geriatric wards (characterized by a large percentage of older patients accompanied by family members). Following an explanation of the aim of the study and the planned procedures for keeping participants’ anonymity, staff members who agreed to participate in the study were asked to complete the self-report questionnaire, then return it in a sealed envelope to a post box in the ward dedicated to this purpose. Altogether 280 questionnaires were distributed to all nurses who work in these units, 193 completed ones were returned, setting the response rate at 69%.

### 2.3. Variables and Measurements

#### 2.3.1. Demographic Characteristics

The following demographic data were collected: gender, age, ethnic origin, marital status, religion, degree of religiousness, education, professional position, employment extent (full-time/part-time), professional seniority and organizational seniority.

#### 2.3.2. Families’ Importance in Nursing Care: Nurses’ Attitudes (FINC-NA) Questionnaire

Perception of the importance of family members in care was examined using the revised version [49] of the Families’ Importance in Nursing Care: Nurses’ Attitudes (FINC-NA) developed by Benzein et al., (2008). The questionnaire consists of 26 items divided into four subscales: 1. Family as a resource in nursing care (Fam-RNC) assesses positive attitudes towards family members and the value of their presence in nursing care (ten items; e.g., “The presence of family members is important to me as a nurse”); 2. Family as a conversational partner (Fam-CP) assesses attitudes towards the importance of acknowledging patient’s family members and having a dialogue with them (eight items; e.g., “I invite family members to speak when planning care”); 3. Family as a burden (Fam-B) assesses negative attitudes towards the presence of family members and time needed to take care of them (four items; e.g., “The presence of family members holds me back in my work”); and 4. Family as its own resource (Fam-OR) assesses attitudes toward family members as having their own resources for coping (four items; e.g., “I encourage families to use their own resources so that they have the optimal possibilities to cope with situations by themselves”). The revised FINC-NA of Saveman et al. (2011) uses a five-point Likert scale (’strongly agree’, “agree”, “neither agree nor disagree”, “disagree” and “strongly disagree”) instead of the 4-point Likert scale used in the original questionnaire. They changed it to increase the variability in the item responses. The questionnaire has a good overall internal reliability of 0.89, and its subscale has a reliability of between 0.71 and 0.86 [49]. This questionnaire was used also in hospitals [50] with an overall reliability of 0.9 and between 0.61 and 0.85 for the subscales. The questionnaire was forward- and backward-translated to Hebrew by experts in the field; however, they had not previously undergone a validation study. For data processing, one average was calculated for the total scale and four averages were calculated for each subscale. In the present study, internal consistency (Cronbach’s alpha) was 0.90 for the total scale and between 0.74 to 0.84 for the four subscales.

#### 2.3.3. Staff Behavior: Involving Family Members in Care

Staff behavior that involves family members in care which is reflected in respect, collaboration and support in the family-centered approach was measured using 15 questions from the Patient Relatives’ Perception of Quality of Geriatric Care questionnaire [51]. The original includes 31 questions divided into 8 sub-scales and has an internal reliability of 0.73 to 0.83. The questions were adapted to express staff behavior in hospitals regarding involvement in care, using the 4 sub-scales that demonstrated the family-centered approach (information, caring process, contact, and participation). Sample questions: “I give the family sufficient information concerning their relative’s medicines;” “I give the family support when they need it.” Each item has five possible answers ranging from 1 = very little or not at all to 5 = to a great extent. The questionnaire was forward- and backward-translated to Hebrew by experts in the field; however, they had not previously undergone a validation study. For data processing, one average was calculated for the scale of staff behavior. In the present study, internal consistency (Cronbach’s alpha) was 0.88.

#### 2.3.4. Perception of Interactions with Family Members

Perception of the interactions with family members include three aspects:

1. Family behaviors towards the staff using the Family Behavior Scale [52], with an internal reliability of 0.69. The questionnaire has four items. For example: “The family treats you with respect,” with five possible answers, from 1 = never to 5 = always. The questionnaire was forward- and backward-translated to Hebrew by experts in the field; however, they have not previously undergone a validation study. In the present study, internal consistency (Cronbach’s alpha) was 0.72.
2. Communication of the family with the staff was examined via eight items based on the short version of the Verbal Abuse Scale (VAS) questionnaire [53] originally developed by Manderino and Banton [54]. The original includes 11 statements and has an internal reliability of 0.86. The short scale includes 8 statements, while the internal validity and reliability testing was not undertaken [53]. Each item has five possible answers, from 1 = very little or not at all to 5 = to a great extent. The aim of the questionnaire is to indicate ineffective communication. Example statement: “Family members speaks to you in a condescending manner.” The questionnaire was forward- and backward-translated to Hebrew by experts in the field; however, they had not previously undergone a validation study. In the present study, internal consistency (Cronbach’s alpha) was 0.95.
3. Conflicts with family members were examined using the Frequency of Interpersonal Conflict Scale [55] (internal reliability 0.86), which includes eight items on different issues around which conflicts may arise, such as personal care, meals, laundry or listening to patient’s needs. For example: “How often do you have arguments or conflicts with family members about meals/food?” Each item has five possible answers, from 1 = never to 5 = every day. The questionnaire was forward- and backward-translated to Hebrew by experts in the field; however, they had not previously undergone a validation study. In the present study, internal consistency (Cronbach’s alpha) was 0.91.

### 2.4. Data Analysis

Analyses were conducted using the IBM SPSS Statistics 25.0. The analysis was calculated on 179 responses. Missing values were less than 0.4% and were not replaced. Analyses were performed in three steps. First, we performed Pearson correlations between the variables. Second, we conducted a multiple-regression analysis to test the contribution of all relationship variables to predicted staff behavior. Finally, we conducted an analysis using a multiple-mediation approach [56]. This analysis ensured unstandardized direct effects, as well as the unique indirect effect of each of the interaction variables (Family behavior, Communication and Conflict) as mediating variables, and the combined overall effect of the mediating variables. Three mediators (Family behavior, Communication and Conflict) were entered into the model simultaneously. The multiple-mediation approach utilizes a bootstrap test, for which we generated 5000 samples, to produce 95% confidence intervals which indicate a significant indirect effect if they do not include 0 [57].

## 3. Results

### 3.1. Participants’ Characteristics

After a total of 14 cases with significant missing data were removed, our final sample included a total of 179 nurses who work in 13 Internal Medicine, Surgical and Geriatric wards. The participants’ characteristics are presented in Table 1.

The majority were women (67%), married (75.4%), with ages ranging from 23 to 66 years (*M* = 40.6, *SD* = 10.5). Thirty-seven percent were Jews, twenty-seven percent were Arab Christians, twenty-six percent were Arab Muslims, and four percent were Druze. Most of the participants had an academic education (53% Bachelor’s degree and 33% Master’s degree). The average number of years of seniority in nursing is 13.8 (*SD* = 10.9), and most respondents work full-time (66.5%). Nineteen percent of study participants were certified nurses with leadership responsibilities, and seventy-nine percent were certified nurses without leadership responsibilities.

### 3.2. Relationship among Study Measures

Correlations among the study measures were explored and are reported in Table 2.

As can be seen, there was a significant correlation between the total FINC-NA scale and its four subscales and staff behavior. The more the nurses perceive the family as a resource, as a conversational partner and as its own resource, and the less they perceive them as a burden, the more positive their behavior. In addition, a significant correlation was found between the Fam-B subscale (Family as a burden) and all the interaction variables (Family behavior, Communication and Conflict). The more negative the communication the higher the level of conflict, and the more negative the perception of the relationship, the more the family is perceived as a burden. Moreover, a significant positive correlation was found between the Fam-OR subscale (Family as its own resource) and the interactions of family behavior variable. The more the nurses feel the family behaves with respect, the more resourceful he or she perceives the family to be. Finally, a significant positive correlation was found between the interactions of family behavior and staff behavior, and a significant negative correlation was found between the interactions conflict and staff behavior. Staff behavior toward a patient’s family is more positive when nurses perceive their relationship with the family as positive and they experience fewer conflicts with family members.

### 3.3. Multiple Regression Analysis Predicting Staff Behavior

A hierarchical multiple linear regression was used to predict staff behavior. In Step 1, the following socio-demographic variables were entered into the regression: age, gender (as dummy variable), years in profession and education. Since education is an ordinal variable, we entered it into the regression as two dummy variables: 1. Bachelor’s degree (1—Bachelor’s degree, 0—Otherwise); 2. Master’s degree (1—Master’s degree, 0—Otherwise). Among the socio-demographic variables that entered in the first step, only the two education variables were significant predictors of staff behavior. Therefore, it was decided not to include in the regression the variables that were not found to be significant.

In Step 2, the interactions variables (Family behavior, Communication and Conflict) and attitudes toward family importance subscales (Fam-RNC, Fam-CP, Fam-B and Fam-OR) were entered into the regression. The results of this analysis are summarized in Table 3.

In step 1, education was a significant predictor of staff behavior (β = 0.31, *p* < 0.01 for Bachelor’s degree and Master’s degree). In Step 2, conflict (β = −0.20, *p* < 0.01), Fam-CP (β = 0.32, *p* < 0.01) and Fam-OR (β = 0.35, *p* < 0.001) were significant predictors of staff behavior. Less conflict and more positive nursing attitudes in terms of the subscales Fam-CP and Fam-OR were associated with higher levels of staff behavior. Family behavior, communication, Fam-RNC and Fam-B did not significantly predict staff behavior. The final model accounted for 43% of the variance in staff behavior (*F*(9, 166) = 14.08, *p* < 0.001).

### 3.4. Interactions as Mediating between FINC-NA and Staff Behavior

To test the mediation models, we used the multiple mediation approach [56]. We examined four models for each of the subscales of FINC-NA as independent variables and staff behavior as a dependent variable. Three mediators (Family behavior, Communication and Conflict) were entered into the models simultaneously. Only the model with the Fam-B as an independent variable was statistically significant. The results of this analysis are summarized in Table 4.

**Figure 2:**
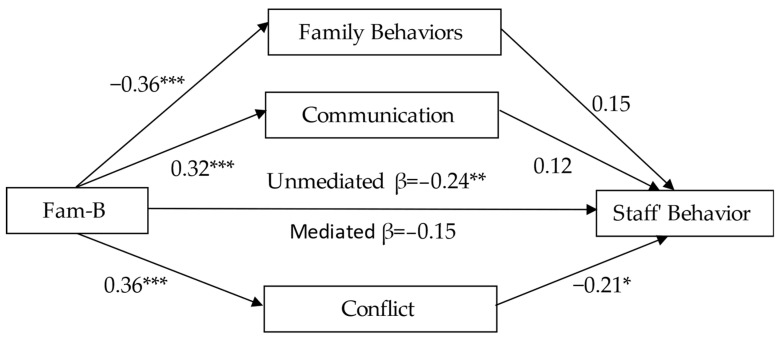
A multiple mediation model of Fam-B and staff behavior through interaction variables (Family Behaviors, Communication and Conflict). Standardized regression coefficients are provided along the paths. * *p* < 0.05, ** *p* < 0.01, *** *p* < 0.001. Abbreviation: Fam-B, family as a burden.

The multiple mediation analyses indicated a significant total effect (*B* = −0.15, SE = 0.05, β = −0.24, *p* < 0.01) between Fam-B and staff behavior. In addition, the direct effect was not significant (*B* = −0.09, SE = 0.05, β = −0.15, *p* > 0.05). As can be seen in Table 4, the total indirect effect of all the mediators was significant–mediation effect = −0.06, *CI* = (−0.12, −0.01). An examination of specific indirect effects indicates that only conflict was a mediator of the relationship between Fam-B and staff behavior (see Table 4). Thus, Fam-B was positively related (β = 0.36) to conflict, which, in turn, was negatively related (β = −0.21) to staff behavior (see Figure 2). The specific indirect effects of Family behavior and communication were not significant, as evidenced by the zero in its confidence interval.

## 4. Discussion

The aim of the present study was to examine the factors predicting nursing staff behavior regarding the involvement of family in the care process of older hospitalized patients. The growing insight that the nurse–family relationship informs the quality of care is reflected in the fact that nurses’ attitudes toward families form an increasingly important research stream (see [58]). This is reflected in many studies that examine encouraging families to become involved in care [59]. These studies also examine the topic of family involvement from different angles, with one of the central aspects being nursing staff’s attitudes regarding the inclusion of family members in patient care [23,26]. These attitudes are some of the important factors in the adoption of family-focused care [32]. In addition, some of the studies indicated that staff attitudes are influenced by personal and well as workplace characteristics [21]. The present study’s goal was to expand existing knowledge in this area. The research model examined the question of whether staff behavior of actively involving the family is related to their perception of the family’s importance in the care process, as well as their interactions with the family. Three mediating variables were examined–family behavior, communication, and conflicts. The findings of the present study have both theoretical and practical contributions to make.

The main finding of the present study points to the importance of staff attitudes towards the involvement of family to staff behavior regarding this issue. Staff perception of the family as conversational partners and as its own resource were found to predict staff behavior of family involvement in patient care, in addition to the perception of interaction with the family as having little conflict and level of staff academic education. This finding supports former findings regarding the influence of staff attitudes regarding the importance of family in care on the encounter between staff and family [22,60]. Moreover, when staff members believed that family presence was important, they tended to involve the family more in the care process [32].

The findings of the present study also support Fishbein and Ajzen’s [61] Reasoned Action Approach, which assumes that people’s attitudes to a certain behavior and their subjective norms towards it lie at the basis of their behavioral intentions and actual behavior. Thus, when the attitudes towards family inclusion are more positive, it is included more in the care process. We may assume that these are also related to the family-centered care norms prevalent at the hospital.

Moreover, all elements of staff’s attitude towards the importance of family in the care process were found to be related to staff behavior with respect to family involvement in care. The more the staff perceives the family as having its own resources, as discourse partners and as a resource in the care process, and perceives them less as a burden, the more their actual behavior will be that of involving the family in the care process. This finding is similar to those of comparable studies regarding the importance of the perception of family as a valued resource as a factor causing them to be invited to participate in the care process and create a positive staff–family relationship [16,22,24].

Staff–family interactions play an important role in staff attitudes, particularly negative ones. Staff perceiving family behavior as disrespectful, its manner of communication as negative and possibly even violent, and experiencing conflicts with it, perceives the family as a burden regarding the care process rather than an important resource. It is well known that negative interactions rob people of various resources, both emotional and concrete. Former research has described staff–family interactions as difficult, conflictual and time-consuming [25,33,34]. In addition, these conflicts may negatively impact the staff’s wellbeing and work performance [20]. In light of the working conditions in hospitals, characterized by overload, tension and lack of human resources, the staff may feel that it needs to “waste” valuable time to come to an understanding with the family and include it in the care process, instead of devoting more time to the patient and the demands of their work. Thus, staff experiencing the interactions with the family as negative will perceive it as a source of further burden, and consequently unimportant or even undesirable as a care partner.

Thus, it is not surprising that we found a connection between perception of the interactions with the family regarding their respectful behavior and conflicts with the family and staff behavior of involving the family in the care process. In a situation in which the staff feels respected, no arguments arise, and there are fewer conflicts with the family, a good relationship is created and a partnership in care is formed [30,31]. Conversely, if the staff feels disrespected and conflicts with the family arise, this may cause feelings of an inability to respond to the family’s needs [38], and no care-related partnership will be formed.

The findings of the present study regarding the influence of conflicts are extremely important, as we understand that conflicts with family members are a significant factor relating to the behavior of involving the family in the care process. The findings of the present study point at the conflict as a factor mediating the connection between the perception of the family as a burden and staff behavior of involving the family in the care process. That is, when a family is perceived as a burden, this leads to further conflict, and the staff will be less inclined to involve the family in the care process. As conflicts with family members are part of the daily routine of the health system [35], this may influence the staff’s willingness to encourage family involvement.

Education was found to be the only factor among the personal and work-related characteristics predicting the behavior of involving the family in the care process. In a similar fashion, education was found in many studies to be related to the perception of the importance of family involvement [21]. Education may promote, beyond knowledge, also awareness and understanding, thus contributing to positive attitudes to the topic. However, a gap was found between perceptions and de facto behavior [26], and this does not necessitate actively inviting the families to become part of the care process [60]. At the same time, this finding points to the importance of education in general, with one of the practical recommendations being to provide knowledge regarding family nursing in general, and the importance of family in the care process specifically, when older people are hospitalized. It is recommended that such education include a reference to the topic of conflicts with families, as well as the practical skills of dealing with them. This will hopefully minimize the conflicts’ influence on staff attitudes and behavior, and promote family-centered care, which sees the family as an inseparable part of the care process, based on the belief that the family is an important resource in caring for hospitalized patients and preserving the patient’s health and emotional wellbeing [8,43].

The present study has several limitations, based on which we make recommendations for further study. First, the study was conducted in one large hospital in northern Israel, with a convenience sample from the nursing staff of that hospital, which limits our ability to generalize the findings. In addition, this type of convenience sample, as well as participants’ self-reporting, may cause biases, such as social desirability. Another limit is that the survey was carried out with questionnaires not previously validated in Hebrew; therefore, it would be advisable to carry out validation studies of the tools before they can be used in larger studies in Israel. It is recommended that the study be expanded to additional hospitals in other parts of the country and that both qualitative and quantitative research methods be used. This will allow the existence of additional factors related to staff attitudes and behavior, such as organization size, ward type, organizational environment and culture, geographical area (periphery or center), and others to be examined. In addition, this will allow us to examine actual staff behavior rather than using self-reports only, as well as deepen our understanding of staff experiences regarding interactions with family members through in-depth interviews. Second, the study was conducted at one point in time (a cross-sectional study), i.e., reflecting the situation at a specific point in time with no reference to other factors which may be connected, such as time or changes in workload or stress. Thus, it is recommended that a longitudinal study be conducted, allowing us to examine the changes in attitudes and behaviors over time.

## 5. Conclusions

The findings of the present study join the existing knowledge in the literature regarding the different positive and negative experiences recounted by the nursing staff regarding the presence and involvement of family members [3]. Moreover, staff behavior in general, and regarding involvement of family in the care process specifically, is motivated by individual perceptions both regarding the importance of this inclusion and regarding staff interactions with the family, mostly its negative aspect, i.e., conflicts. Much has been written about the fact that staff–family relationships can be difficult, and the interactions between the two may cause tension and conflicts [30,35,36] and lead to a lack of cooperation. The present study offers a practical contribution related to the existence of conflicts as a central factor of relationships in general and those necessitating cooperation and reciprocity specifically. As we witness the implications of the population’s aging and the large percentage of older hospitalized patients, it is safe to assume that staff members will encounter more and more family members accompanying such patients. The rise in hospitalization among the 75+ age group, where the family fulfills the role of central mediating agent during the hospitalization period, emphasizes the need to develop tools for nurses and medical teams, maximizing family involvement and assistance. This study supports the work of researchers who stated that education may assist nurses wishing to include family members in the care process but need practical suggestions for action [62]. The study suggests two central elements to be taken into account: communication with family members and conflicts. In light of the importance of staff–family cooperation in an effort to promote quality of care of the hospitalized older patient [28], it is recommended that emphasis be placed on different training and education processes, including both initial nursing studies and ongoing studies throughout one’s career. It is recommended that these studies focus on two central topics—the importance of family in the care process and staff–family communication—while emphasizing conflicts, their perception and ways of dealing with them. The aim of these education processes is to provide the staff not only with knowledge, but also with practical skills that will contribute to their ability to manage conflictual situations with families. This will contribute to both the quality of relationship with the family and the quality of patient care.

## Figures and Tables

**Table 1:** Sample Demographic and Professional Characteristics (*N* = 179).

		*N*	%	*M*	*SD*
Gender ^a^	Female	120	67.00%		
	Male	58	32.40%		
Age ^b^	23–35	63	35.20%	40.6	10.5
	36–50	67	37.40%		
	51 and older	35	19.60%		
Marital Status ^c^	Married	135	75.40%		
	Single	27	15.10%		
	Divorced	12	6.70%		
	Widowed	3	1.70%		
Religion ^d^	Jewish	66	36.90%		
	Muslim	46	25.70%		
	Christian	49	27.40%		
	Druze	7	3.90%		
Education ^c^	Certificate	23	12.80%		
	Bachelor’s degree	95	53.10%		
	Master’s degree	59	33.00%		
Professional position ^e^	Certified nurse no leadership responsibilities	141	78.80%		
	Certified nurse with leadership responsibilities	34	19.00%		
Years in profession	Range = (1–45)			13.8	10.9
Job percentages ^e^	Full time job	119	66.50%		
	Part time job	56	31.30%		

Abbreviations: *M,* Mean; *SD,* standard deviation. ^a^ Missing: 1 (0.6%); ^b^ Missing: 14 (7.8%); ^c^ Missing: 2 (1.1%); ^d^ Missing: 11 (6.1%); ^e^ Missing: 4 (2.2%).

**Table 2:** Pearson Correlations between Study Measures (*N* = 179).

	1	2	3	4	5	6	7	8
1. Staff Behavior	1							
2. Interactions–Family Behaviors	0.20 **	1						
3. Interactions-Communication	−0.11	−0.60 ***	1					
4. Interactions-Conflict	−0.26 ***	−0.32 ***	0.45 ***	1				
5. Fam-RNC	0.41 ***	0.12	−0.07	0.02	1			
6. Fam-CP	0.50 ***	−0.01	0.00	0.03	0.72 ***	1		
7. Fam-B	−0.24 **	−0.36 ***	0.32 ***	0.36 ***	−0.13 *	−0.06	1	
8. Fam-OR	0.56 ***	0.20 **	−0.06	−0.04	0.65 ***	0.69 ***	−0.17 *	1
9. FINC-NA (total)	0.55 ***	0.18 **	−0.12	−0.08	0.90 ***	0.87 ***	−0.36 ***	0.81 ***

* *p* < 0.05, ** *p* < 0.01, *** *p* < 0.001. Abbreviations: Fam-RNC, family as a resource in nursing care; Fam-CP, family as a conversational partner; Fam-B, family as a burden; Fam-OR, family as own resource; FINC-NA, Families’ Importance in Nursing Care–Nurses’ Attitudes.

**Table 3:** Hierarchical Multiple Linear Regression Analysis for Predicting Staff Behavior (*N* = 177).

Predictor Variable	*B*	SE	β	*t*	*p*
Step 1: (Constant)	3.74	0.11		35.09	<0.001
Bachelor’s degree	0.33	0.12	0.31	2.75	**0.007**
Master’s degree	0.34	0.13	0.31	2.67	**0.008**
Step 1: (Constant)	2.38	0.38		6.27	<0.001
Bachelor’s degree	0.22	0.1	0.21	2.24	**0.026**
Master’s degree	0.16	0.1	0.15	1.57	0.117
Interactions-Family Behaviors	0.07	0.07	0.08	1.02	0.309
Interactions-Communication	0.05	0.05	0.08	0.99	0.323
Interactions-Conflict	−0.12	0.04	−0.20	−2.84	**0.005**
Fam-RNC	−0.07	0.08	−0.08	−0.89	0.374
Fam-CP	0.26	0.08	0.32	3.3	**0.001**
Fam-B	−0.05	0.04	−0.09	−1.31	0.191
Fam-OR	0.26	0.07	0.35	3.89	**<0.001**

Abbreviations: *B*, Unstandard coefficient; SE, Standard Error; β, Standard coefficient; Fam-RNC, family as a resource in nursing care; Fam-CP, family as a conversational partner; Fam-B, family as a burden; Fam-OR, family as own resource.

**Table 4:** Indirect Effects of Fam-B on Staff Behavior through Interaction Variables (*N* = 178).

Mediator	Effect	SE	95% BC CI ^‡^
Total	**−0.06**	**0.03**	**[−0.12, −0.01]**
Family Behaviors	−0.03	0.02	[−0.08, 0.01]
Communication	0.02	0.03	[−0.03, 0.08]
Conflict	**−0.05**	**0.02**	**[−0.10, −0.01]**

^‡^ Boldface font highlights a significant effect as determined by the 95% bias-corrected confidence interval (95% BC CI). Abbreviation: SE, Standard Error.

## Data Availability

The data presented in this study are available upon request from the corresponding author.
